# Burden of non-rheumatic valvular heart disease globally and in China from 1990 to 2021: a systematic analysis for the global burden of disease study 2021

**DOI:** 10.3389/fcvm.2025.1641448

**Published:** 2025-11-20

**Authors:** Wen Chen, Jiyong Wei, Xiaoyu Zheng, Yanni Lan, Baoshi Zheng

**Affiliations:** 1 Department of Cardiothoracic Surgery, The First Affliated Hospital of Guangxi Medical University, Nanning, Guangxi, China; 2 Department of Thoracic and Cardiovascular Surgery, The First People’s Hospital of Nanning, The Fifth Affiliated Hospital of Guangxi Medical University, Nanning, Guangxi, China; 3 Department of Orthopedic Surgery, The First People’s Hospital of Nanning, The Fifth Affiliated Hospital of Guangxi Medical University, Nanning, Guangxi, China; 4 Department of Pharmacy, The People’s Hospital of Guangxi Zhuang Autonomous Region & Guangxi Academy of Medical Sciences, Nanning, Guangxi, China

**Keywords:** valvular heart disease, global, China, disease burden, GBD

## Abstract

**Background:**

Non-rheumatic valvular heart disease (NRVHD), driven by aging populations and epidemiological transitions, has become the dominant form of valvular heart disease globally, yet its burden trends and demographic disparities remain undercharacterized. This study quantifies the global and Chinese burden of NRVHD from 1990 to 2021, analyzing age-sex disparities and temporal trends.

**Methods:**

Using data from the Global Burden of Disease (GBD) 2021 Study. First, the number of incidence, prevalence, deaths, and disability-adjusted life years (DALYs) cases, along with their corresponding age-standardized rates (ASRs), were reported globally and in China, stratified by different sub-types in 2021. These sub-types included sex and age groups. Second, to explore the temporal trend of the disease burden, data from 1990 to 2021 were analyzed both globally and by sub-types. The estimated annual percentage change (EAPC) value was calculated using a linear regression model.

**Results:**

In 2021, NRVHD caused 2,206,928 global incidence cases (ASR: 25.0/100,000) and 28.4 million prevalence cases (ASR: 335.3/100,000), with 181,078 deaths (ASR: 2.31/100,000) and 3.24 million DALYs (ASR: 39.7/100,000). China accounted for 292,215 incidence cases (ASR: 12.8/100,000) and 3.28 million prevalence cases (ASR: 156.2/100,000), demonstrating lower deaths cases (ASDR: 0.13 vs. global 2.31/100,000) but accelerated burden growth. From 1990 to 2021, global incidence rose 129%, while China's surged 220%. Age-standardized deaths rate declined globally and in China, contrasting with rising prevalence. Males bore higher burdens globally and in China, with elderly populations disproportionately affected.

**Conclusion:**

NRVHD burden has escalated globally, characterized by rising morbidity amid declining mortality, a paradox amplified in China. While therapeutic advances and hypertension control contributed to mortality reductions, persistent sex-age disparities and rural-urban inequities demand targeted strategies. China's rapid epidemiological transition underscores the urgency of integrating primary prevention, equitable technology access, and enhanced surveillance to address aging-related valvulopathies.

## 1 Introduction

Valvular heart disease (VHD) remains a significant contributor to global cardiovascular morbidity and mortality, with non-rheumatic valvular heart disease (NRVHD) emerging as an increasingly critical public health concern in the context of aging populations and epidemiological transitions. While rheumatic heart disease (RHD) has historically dominated VHD burden in low-income regions, NRVHD, encompassing degenerative, congenital, ischemic, and other non-infectious etiologies, now accounts for over 65% of global VHD cases, particularly in high- and middle-income countries (1). This shift parallels demographic aging, as degenerative aortic valve disease prevalence rises exponentially after age 65 (2), and increased survival from cardiovascular comorbidities like hypertension and coronary artery disease (3).

The Global Burden of Disease (GBD) Study 2021 provides critical insights into this evolving landscape, building upon previous analyses that identified VHD as responsible for 12.6 million prevalent cases and 262,000 deaths globally in 2017 (4). However, prior GBD reports have not systematically differentiated NRVHD from RHD in national-level assessments, potentially obscuring distinct risk factor profiles and intervention needs. This gap is particularly relevant for China, where rapid population aging [projected 26% population ≥60 years by 2030 (5)] coincides with dramatic declines in RHD prevalence from 1.19/1000 in 1990 to 0.32/1000 in 2019 (6), while degenerative VHD hospitalizations increased 4.8-fold between 2003−2016 (7).

Recent studies highlight the growing NRVHD burden in China, with aortic stenosis prevalence tripling among adults ≥75 years from 2001−2015 (8), and mitral regurgitation affecting 19.3% of community-dwelling elderly (9). Yet these regional analyses lack integration with global trends and standardized disability metrics. The GBD framework enables comparative assessment through disability-adjusted life years (DALYs), which capture both premature mortality and functional impairment, critical for diseases like NRVHD where interventions often prioritize quality-of-life preservation over survival (10).

This study leverages GBD 2021 data to: (1) Quantify NRVHD burden globally and in China from 1990−2021; (2) Analyze age-sex disparities. Our findings aim to inform health policies addressing the dual challenges of aging-related cardiovascular disease and persistent health disparities in VHD management.

## 2 Methods

### 2.1 Data sources

Epidemiological data were extracted from the GBD 2021 database, which aggregates input sources across 204 countries and territories. Key data references included:

Systematic reviews and population-based studies: A comprehensive literature search (1990–2021) was performed across PubMed, EMBASE, China National Knowledge Infrastructure (CNKI), and regional databases to identify studies reporting NRVHD incidence, prevalence, and mortality. Studies were included if they provided echocardiographically confirmed diagnoses based on international guidelines (≥moderate aortic stenosis defined by peak velocity ≥3 m/s) (11).

Vital registration systems: Death certificates and hospital records coded with ICD-10 classifications (I05–I08, I34–I38) were obtained from national health agencies, including the Chinese Center for Disease Control and Prevention (CDC) and WHO Mortality Database. NRVHD cases were defined as ICD-10 I34–I38, excluding rheumatic heart disease (I05–I08).

Disease registries: Subnational data for China were supplemented by the China Cardiovascular Association (CCA) Valve Disease Registry (2015–2020), covering 31 provinces with 28,543 NRVHD cases (12).

### 2.2 Data processing and modeling

Case definitions aligned with GBD 2021 cause hierarchies, excluding post-rheumatic cases (GBD cause code 294). The DisMod-MR 2.1 Bayesian meta-regression tool was employed to synthesize heterogeneous data, adjusting for covariates including: Echocardiography access, Ambient particulate matter pollution (PM_2.5_) exposure, and Comorbidity index (aggregating hypertension, diabetes, and coronary artery disease). Covariate selection adhered to the GBD 2021 hierarchy (13). Age-standardized rates (ASRs) were calculated using the GBD 2021 global reference population, with uncertainty intervals (UIs) derived from 1,000 posterior draws of the model.

### 2.3 Statistical analysis

First, the description of the disease burden was carried out. In 2021, the number of incidence, prevalence, deaths, and DALYs of NRVHD, along with their corresponding age-standardized rates (ASRs), were reported globally and in China, stratified by different sub-types. These sub-types included sex and age groups.

Second, to explore the temporal trend of the disease burden, data from 1990 to 2021 were analyzed both globally and by sub-types. “The estimated annual percentage change (EAPC) value was calculated by fitting a log-linear regression model to the ASRs” (Equations 1, 2) (45). Specifically, the model was defined as:

ln(ASR)=α+β×year (1)

where *α* is the intercept and *β* represents the slope coefficient. The EAPC was then derived as:

EAPC=[exp(β)−1]×100% (2)

Statistical significance was determined when the *P*-value was less than 0.05. All statistical analyses, including database construction, collation, and analysis, were performed using the R (version 4.0.2) software.

## 3 Results

### 3.1 Global and national burden of NRVHD in 2021

In 2021, NRVHD accounted for substantial global disease burden. Globally, there were 2,206,928 incidence cases (95% UI: 2,048,267–2,375,325), corresponding to an age-standardized incidence rate (ASIR) of 25.0 per 100,000 population (95% UI: 23.17–26.92). The prevalence reached 28,389,034 cases (95% UI: 26,323,458–30,585,670), with an age-standardized prevalence rate (ASPR) of 335.29 per 100,000 (95% UI: 311.34–361.05). NRVHD-related deaths totaled 181,078 (95% UI: 155,364–195,717), yielding an age-standardized death rate (ASDR) of 2.31 per 100,000 (95% UI: 1.96–2.50). The DALYs burden stood at 3,238,185 (95% UI: 2,934,104–3,594,474), with an age-standardized DALYs rate (ASDAR) of 39.72 per 100,000 (95% UI: 35.79–44.14) (Tables 1–4).

**Table 1:** The number of incidence cases and the age-standardized incidence rate attributable to non-rheumatic valvular heart disease globally and in China in 1990 and 2021, and its trends from 1990 to 2021 globally.

Region/Subgroup	Number of incidence cases (95% UI) in 1990	The age-standardized incidence rate/100,000 (95% UI) in 1990	Number of incidence cases (95% UI) in 2021	The age-standardized incidence rate/100,000 (95% UI) in 2021	EAPC (95% CI)
Global	963,242 (885,978–1,047,599)	23.9 (21.98–26)	2,206,928 (2,048,267–2,375,325)	25 (23.17–26.92)	0.34 (0.28–0.41)
Sex in global					
Female	391,873 (357,548–430,818)	18.26 (16.67–20.07)	879,064 (810,710–949,106)	18.85 (17.39–20.34)	0.3 (0.23–0.37)
Male	571,370 (527,414–620,269)	30.04 (27.84–32.59)	1,327,864 (1,235,519–1,427,185)	31.64 (29.38–34)	0.37 (0.31–0.43)
Age in global					
15–19 years	1,191 (938–1,516)	0.23 (0.18–0.29)	1,372 (1,073–1,756)	0.22 (0.17–0.28)	−0.18 (−0.28 to 0.07)
20–24 years	3,467 (2,734–4,399)	0.7 (0.56–0.89)	4,071 (3,189–5,203)	0.68 (0.53–0.87)	−0.16 (−0.22 to 0.1)
25–29 years	5,661 (4,480–7,153)	1.28 (1.01–1.62)	6,928 (5,443–8,845)	1.18 (0.93–1.5)	−0.14 (−0.23 to 0.04)
30–34 years	7,485 (5,936–9,444)	1.94 (1.54–2.45)	10,158 (7,995–12,956)	1.68 (1.32–2.14)	-0.32 (−0.51–0.13)
35–39 years	8,839 (7,017–11,146)	2.51 (1.99–3.16)	12,696 (10,010–16,178)	2.26 (1.78–2.88)	-0.35 (−0.54 to 0.17)
40–44 years	15,135 (11,908–19,011)	5.28 (4.16–6.64)	23,582 (18,540–29,852)	4.71 (3.71–5.97)	−0.48 (−0.58 to 0.37)
45–49 years	22,859 (15,169–31,661)	9.84 (6.53–13.64)	41,858 (27,818–59,948)	8.84 (5.87–12.66)	−0.52 (−0.6 to 0.43)
50–54 years	57,175 (47,100–69,151)	26.9 (22.16–32.53)	100,857 (82,547–121,164)	22.67 (18.55–27.23)	−0.5 (−0.58 to 0.42)
55–59 years	103,262 (80,312–129,858)	55.76 (43.36–70.12)	191,623 (151,879–241,254)	48.42 (38.38–60.96)	−0.28 (−0.37 to 0.2)
60–64 years	183,219 (162,238–207,656)	114.08 (101.01–129.29)	365,854 (328,460–406,635)	114.31 (102.63–127.05)	0.22 (0.12–0.32)
65–69 years	250,099 (219,637–286,782)	202.33 (177.69–232.01)	582,588 (519,507–653,562)	211.2 (188.33–236.93)	0.49 (0.36–0.61)
70–74 years	172,619 (150,519–197,178)	203.89 (177.79–232.9)	494,143 (441,640–551,174)	240.06 (214.56–267.77)	0.64 (0.56–0.71)
75–79 years	83,526 (65,348–101,745)	135.69 (106.16–165.29)	212,657 (174,215–251,894)	161.25 (132.1–191)	0.75 (0.69–0.82)
80–84 years	31,878 (20,922–44,107)	90.11 (59.14–124.68)	99,507 (69,201–129,526)	113.61 (79.01–147.89)	0.85 (0.73–0.97)
85–89 years	11,895 (7,511–17,678)	78.72 (49.7–116.99)	39,952 (27,440–55,934)	87.38 (60.02–122.34)	0.67 (0.44–0.89)
90–94 years	3,718 (1,990–5,885)	86.77 (46.43–137.33)	14,073 (7,826–21,970)	78.67 (43.75–122.81)	0.36 (−0.11 to 0.83)
95+ years	1,214 (501–2,286)	119.26 (49.19–224.58)	5,008 (2,184–9,165)	91.88 (40.07–168.15)	−0.11 (−0.75–0.54)
China	91,321 (85,425–97,428)	10.27 (9.63–10.97)	292,215 (275,208–310,319)	12.75 (12.01–13.54)	0.92 (0.83–1.01)
Sex in China					
Female	31,855 (29,802–34,153)	7.13 (6.68–7.67)	101,018 (94,641–108,048)	8.65 (8.1–9.26)	0.82 (0.73–0.91)
Male	59,466 (55,585–63,254)	13.48 (12.63–14.36)	191,196 (180,067–202,860)	16.96 (16.02–18)	0.99 (0.89–1.09)
Age in China					
15–19 years	81 (57–109)	0.06 (0.05–0.09)	65 (45–88)	0.09 (0.06–0.12)	1.01 (0.94–1.07)
20–24 years	257 (181–344)	0.19 (0.14–0.26)	199 (139–271)	0.27 (0.19–0.37)	1.15 (1.11–1.19)
25–29 years	363 (257–485)	0.33 (0.23–0.44)	401 (281–545)	0.46 (0.33–0.63)	1.16 (1.1–1.22)
30–34 years	416 (295–555)	0.47 (0.33–0.63)	767 (536–1,045)	0.63 (0.44–0.86)	1.07 (0.96–1.17)
35–39 years	542 (384–726)	0.59 (0.42–0.79)	863 (604–1,175)	0.81 (0.57–1.11)	1.02 (0.92–1.13)
40–44 years	1,168 (899–1,514)	1.74 (1.34–2.26)	1,819 (1,373–2,461)	1.99 (1.5–2.69)	0.16 (0.02–0.3)
45–49 years	2,017 (1,355–2,849)	3.91 (2.63–5.52)	4,504 (2,836–6,746)	4.08 (2.57–6.12)	−0.15 (−0.34 to 0.03)
50–54 years	5,241 (4,444–6,255)	10.98 (9.31–13.11)	13,247 (11,120–15,839)	10.96 (9.2–13.1)	−0.16 (−0.25 to 0.07)
55–59 years	9,945 (7,745–12,270)	22.93 (17.86–28.29)	24,748 (19,869–30,270)	22.51 (18.07–27.53)	−0.15 (−0.24 to 0.06)
60–64 years	18,945 (17,245–20,681)	53.61 (48.8–58.52)	47,980 (44,302–51,969)	65.72 (60.68–71.19)	0.82 (0.72–0.92)
65–69 years	27,651 (24,917–30,428)	101.35 (91.33–111.53)	102,023 (93,366–111,020)	133.01 (121.72–144.74)	1.22 (1.07–1.37)
70–74 years	18,236 (16,333–20,177)	96.91 (86.8–107.22)	68,590 (62,929–74,299)	128.7 (118.07–139.41)	1.31 (1.14–1.48)
75–79 years	5,092 (4,465–5,930)	44.74 (39.23–52.11)	19,207 (16,893–21,938)	57.99 (51.01–66.24)	1.2 (1.06–1.33)
80–84 years	986 (640–1,456)	18.62 (12.08–27.48)	4,704 (3,076–6,642)	23.77 (15.54–33.56)	0.69 (0.61–0.77)
85–89 years	309 (193–458)	18.32 (11.46–27.15)	2,186 (1,368–3,247)	22.95 (14.36–34.08)	0.48 (0.35–0.61)
90–94 years	61 (37–94)	20.01 (12.13–30.72)	725 (425–1,140)	24.72 (14.5–38.87)	0.33 (0.17–0.49)
95+ years	9 (4–17)	23.28 (10.07–42.55)	189 (78–354)	29.53 (12.2–55.41)	0.33 (0.15–0.51)

EAPC, estimated annual percentage change; UI, uncertainty interval; CI, confidence interval.

**Table 2:** The number of prevalence cases and the age-standardized prevalence rate attributable to non-rheumatic valvular heart disease globally and in China in 1990 and 2021, and its trends from 1990 to 2021 globally.

Region/Subgroup	Number of prevalence cases (95% UI) in 1990	The age-standardized prevalence rate/100,000 (95% UI) in 1990	Number of prevalence cases (95% UI) in 2021	The age-standardized prevalence rate/100,000 (95% UI) in 2021	EAPC (95% CI)
Global	11,670,353 (10,755,209–12,710,746)	318.94 (294.11–345.46)	28,389,034 (26,323,458–30,585,670)	335.29 (311.34–361.05)	0.31 (0.25–0.38)
Sex in global					
Female	4,940,546 (4,528,844–5,395,246)	240.65 (220.44–262.77)	11,562,832 (10,702,857–12,514,445)	247.42 (228.96–267.83)	0.23 (0.16–0.3)
Male	6,729,808 (6,233,012–7,304,796)	422.15 (390.75–456.35)	16,826,202 (15,662,022–18,068,827)	442.45 (412.66–474.66)	0.31 (0.26–0.37)
Age in global					
15–19 years	2,726 (2,202–3,292)	0.52 (0.42–0.63)	4,043 (3,218–4,978)	0.65 (0.52–0.8)	0.56 (0.47–0.65)
20–24 years	12,713 (10,186–16,020)	2.58 (2.07–3.26)	15,402 (12,248–19,410)	2.58 (2.05–3.25)	−0.06 (−0.13 to 0)
25–29 years	31,992 (25,564–40,525)	7.23 (5.78–9.16)	39,031 (30,730–49,664)	6.63 (5.22–8.44)	−0.13 (−0.24 to 0.03)
30–34 years	57,804 (46,218–73,365)	15 (11.99–19.03)	77,437 (61,083–98,716)	12.81 (10.1–16.33)	−0.36 (−0.56 to 0.15)
35–39 years	87,641 (69,875–111,344)	24.88 (19.84–31.61)	124,358 (98,114–158,685)	22.17 (17.49–28.29)	−0.39 (−0.59 to 0.2)
40–44 years	125,954 (103,390–155,218)	43.97 (36.09–54.18)	191,657 (154,919–235,215)	38.31 (30.97–47.02)	−0.51 (−0.62 to 0.39)
45–49 years	184,291 (150,673–221,097)	79.37 (64.89–95.22)	334,119 (271,645–405,149)	70.56 (57.37–85.56)	−0.52 (−0.62 to 0.41)
50–54 years	344,092 (277,049–421,142)	161.87 (130.33–198.12)	622,256 (500,899–765,869)	139.86 (112.58–172.14)	−0.48 (−0.55 to 0.4)
55–59 years	672,061 (571,075–792,107)	362.88 (308.36–427.7)	1,269,535 (1,081,583–1,492,707)	320.81 (273.31–377.21)	−0.27 (−0.36 to 0.19)
60–64 years	1,259,734 (1,076,853–1,482,694)	784.35 (670.48–923.17)	2,345,094 (2,009,177–2,709,405)	732.73 (627.77–846.56)	−0.13 (−0.2 to 0.07)
65–69 years	1,948,034 (1,759,060–2,174,251)	1,575.96 (1,423.08–1,758.97)	4,213,557 (3,837,009–4,653,442)	1,527.52 (1,391.02–1,686.99)	0.11 (0.03–0.2)
70–74 years	2,205,492 (2,002,891–2,425,447)	2,605.08 (2,365.77–2,864.89)	5,925,109 (5,444,791–6,416,578)	2,878.51 (2,645.17–3,117.28)	0.37 (0.3–0.44)
75–79 years	2,273,486 (2,083,060–2,495,596)	3,693.39 (3,384.03–4,054.22)	5,121,129 (4,754,044–5,527,784)	3,883.04 (3,604.7–4,191.39)	0.42 (0.32–0.53)
80–84 years	1,505,914 (1,360,954–1,658,976)	4,256.89 (3,847.12–4,689.56)	4,236,098 (3,893,011–4,584,610)	4,836.66 (4,444.94–5,234.59)	0.53 (0.44–0.63)
85–89 years	698,510 (620,992–777,252)	4,622.51 (4,109.52–5,143.59)	2,508,105 (2,287,313–2,735,199)	5,485.58 (5,002.68–5,982.27)	0.78 (0.66–0.9)
90–94 years	209,373 (183,155–237,543)	4,885.97 (4,274.15–5,543.35)	1,058,124 (948,176–1,166,387)	5,914.82 (5,300.22–6,520)	0.9 (0.83–0.97)
95+ years	50,538 (42,690–59,329)	4,964.03 (4,193.14–5,827.53)	303,981 (262,355–347,365)	5,577.31 (4,813.57–6,373.31)	0.57 (0.43–0.72)
China	929,466 (865,891–1,000,057)	127.84 (119.91–136.94)	3,277,574 (3,093,730–3,482,108)	156.24 (147.74–165.63)	0.88 (0.79–0.96)
Sex in China					
Female	342,957 (319,757–369,245)	89.79 (83.96–96.26)	1,182,215 (1,101,983–1,266,498)	106.53 (99.71–114.04)	0.77 (0.68–0.86)
Male	586,509 (546,272–630,775)	175.69 (164.86–187.23)	2,095,359 (1,981,134–2,221,324)	214.59 (202.99–227.25)	0.89 (0.8–0.98)
Age in China					
15–19 years	193 (146–246)	0.15 (0.12–0.19)	193 (146–243)	0.26 (0.2–0.33)	1.71 (1.59–1.82)
20–24 years	933 (664–1,250)	0.71 (0.5–0.95)	774 (564–1,019)	1.06 (0.77–1.39)	1.36 (1.34–1.39)
25–29 years	1,999 (1,419–2,710)	1.82 (1.29–2.47)	2,310 (1,646–3,089)	2.67 (1.9–3.57)	1.34 (1.28–1.4)
30–34 years	3,104 (2,200–4,212)	3.52 (2.49–4.77)	5,957 (4,191–8,024)	4.92 (3.46–6.62)	1.22 (1.11–1.33)
35–39 years	5,154 (3,623–7,019)	5.64 (3.97–7.68)	8,578 (6,033–11,569)	8.09 (5.69–10.92)	1.18 (1.07–1.29)
40–44 years	6,828 (5,273–8,715)	10.18 (7.86–12.99)	12,542 (9,483–16,168)	13.7 (10.36–17.66)	0.88 (0.82–0.93)
45–49 years	12,111 (9,495–15,271)	23.46 (18.39–29.58)	30,575 (23,914–39,845)	27.71 (21.68–36.12)	0.38 (0.28–0.49)
50–54 years	26,607 (21,430–33,163)	55.77 (44.92–69.51)	72,584 (56,913–94,583)	60.06 (47.09–78.26)	0.09 (−0.01 to 0.18)
55–59 years	60,026 (51,394–70,491)	138.41 (118.5–162.54)	155,495 (132,473–183,989)	141.43 (120.49–167.35)	−0.04 (−0.15 to 0.06)
60–64 years	109,072 (95,308–125,875)	308.66 (269.71–356.21)	247,041 (218,500–279,726)	338.39 (299.29–383.16)	0.18 (0.12–0.25)
65–69 years	185,810 (171,924–201,244)	681.07 (630.18–737.65)	623,692 (581,449–672,142)	813.12 (758.05–876.29)	0.7 (0.65–0.76)
70–74 years	224,073 (208,599–240,592)	1,190.76 (1,108.53–1,278.54)	796,334 (744,685–851,945)	1,494.16 (1,397.25–1,598.5)	1.01 (0.89–1.13)
75–79 years	173,937 (161,498–186,626)	1,528.35 (1,419.05–1,639.84)	634,303 (594,845–679,192)	1,915.23 (1,796.09–2,050.77)	1.14 (0.98–1.3)
80–84 years	85,748 (79,899–92,334)	1,618.77 (1,508.35–1,743.1)	410,271 (384,897–438,967)	2,072.93 (1,944.72–2,217.92)	1.17 (1.03–1.31)
85–89 years	28,011 (26,016–30,278)	1,660.56 (1,542.28–1,794.92)	202,161 (188,943–216,402)	2,122.26 (1,983.5–2,271.76)	1.07 (0.94–1.19)
90–94 years	5,224 (4,829–5,667)	1,702.76 (1,573.94–1,847.02)	61,415 (56,878–66,363)	2,094.67 (1,939.9–2,263.4)	0.91 (0.78–1.03)
95+ years	637 (579–704)	1,572.73 (1,429.45–1,738.27)	13,350 (12,227–14,833)	2,088.93 (1,913.12–2,320.95)	0.97 (0.89–1.05)

EAPC, estimated annual percentage change; UI, uncertainty interval; CI, confidence interval.

**Table 3:** The number of deaths cases and the age-standardized deaths rate attributable to non-rheumatic valvular heart disease globally and in China in 1990 and 2021, and its trends from 1990 to 2021 globally.

Region/Subgroup	Number of deaths cases (95% UI) in 1990	The age-standardized deaths rate/100,000 (95% UI) in 1990	Number of deaths cases (95% UI) in 2021	The age-standardized deaths rate/100,000 (95% UI) in 2021	EAPC (95% CI)
Global	82,630 (76,087–88,392)	2.66 (2.4–2.84)	181,078 (155,364–195,717)	2.31 (1.96–2.5)	−0.37 (−0.46 to 0.27)
Sex in global					
Female	48,450 (43,197–52,543)	2.66 (2.34–2.88)	106,463 (86,729–118,494)	2.25 (1.84–2.5)	−0.45 (−0.54 to 0.36)
Male	34,180 (32,258–36,282)	2.55 (2.36–2.69)	74,615 (67,022–79,013)	2.32 (2.06–2.47)	−0.21 (−0.34 to 0.09)
Age in global					
15–19 years	551 (418–700)	0.11 (0.08–0.13)	580 (479–680)	0.09 (0.08–0.11)	−0.57 (−0.67 to 0.47)
20–24 years	625 (477–771)	0.13 (0.1–0.16)	721 (603–843)	0.12 (0.1–0.14)	−0.39 (−0.5–0.28)
25–29 years	702 (569–853)	0.16 (0.13–0.19)	801 (688–918)	0.14 (0.12–0.16)	−0.47 (−0.55 to 0.39)
30–34 years	794 (672–941)	0.21 (0.17–0.24)	995 (875–1,115)	0.16 (0.14–0.18)	−0.66 (−0.78 to 0.54)
35–39 years	1,007 (867–1,181)	0.29 (0.25–0.34)	1,320 (1,160–1,488)	0.24 (0.21–0.27)	−0.7 (−0.88 to 0.52)
40–44 years	1,245 (1,095–1,433)	0.43 (0.38–0.5)	1,765 (1,590–1,950)	0.35 (0.32–0.39)	−0.91 (−1.12 to 0.7)
45–49 years	1,495 (1,340–1,715)	0.64 (0.58–0.74)	2,203 (1,994–2,466)	0.47 (0.42–0.52)	−1.22 (−1.37 to 1.07)
50–54 years	2,172 (1,971–2,454)	1.02 (0.93–1.15)	3,121 (2,848–3,371)	0.7 (0.64–0.76)	−1.3 (−1.45 to 1.16)
55–59 years	3,352 (3,045–3,745)	1.81 (1.64–2.02)	4,940 (4,526–5,388)	1.25 (1.14–1.36)	−1.15 (−1.36 to 0.94)
60–64 years	4,948 (4,604–5,369)	3.08 (2.87–3.34)	6,643 (6,162–7,127)	2.08 (1.93–2.23)	−1.38 (−1.58 to 1.17)
65–69 years	7,013 (6,564–7,524)	5.67 (5.31–6.09)	9,525 (8,778–10,250)	3.45 (3.18–3.72)	−1.57 (−1.77 to 1.37)
70–74 years	8,844 (8,267–9,454)	10.45 (9.76–11.17)	13,792 (12,800–14,673)	6.7 (6.22–7.13)	−1.61 (−1.81 to 1.42)
75–79 years	13,185 (12,436–13,928)	21.42 (20.2–22.63)	17,722 (16,183–18,976)	13.44 (12.27–14.39)	−1.53 (−1.63 to 1.42)
80–84 years	15,070 (13,562–16,067)	42.6 (38.34–45.42)	27,333 (23,407–29,619)	31.21 (26.73–33.82)	−0.99 (−1.11 to 0.87)
85–89 years	12,535 (10,665–13,606)	82.96 (70.57–90.04)	35,795 (28,385–39,759)	78.29 (62.08–86.96)	0.02 (−0.15 to 0.19)
90–94 years	6,732 (5,505–7,450)	157.09 (128.47–173.85)	34,158 (26,116–38,398)	190.94 (145.99–214.64)	0.88 (0.73–1.03)
95 + years	2,360 (1,778–2,667)	231.79 (174.61–261.98)	19,661 (13,889–22,783)	360.74 (254.84–418.01)	1.47 (1.33–1.61)
China	1,535 (1,151–1,862)	0.23 (0.17–0.28)	2,458 (1,952–3,079)	0.13 (0.11–0.17)	−2.28 (−2.6 to 1.97)
Sex in China					
Female	722 (412–992)	0.21 (0.12–0.28)	1,063 (736–1,557)	0.1 (0.07–0.15)	−2.79 (−3.12 to 2.46)
Male	813 (623–1,012)	0.27 (0.2–0.33)	1,395 (1,091–1,763)	0.17 (0.14–0.22)	−1.81 (−2.13 to 1.49)
Age in China					
15–19 years	41 (29–52)	0.03 (0.02–0.04)	8 (6–11)	0.01 (0.01–0.01)	−4.39 (−4.8 to 3.99)
20–24 years	51 (36–64)	0.04 (0.03–0.05)	14 (11–18)	0.02 (0.01–0.03)	−3.14 (−3.55 to 2.72)
25–29 years	38 (27–49)	0.03 (0.02–0.04)	17 (14–22)	0.02 (0.02–0.02)	−2.46 (−2.76 to 2.17)
30–34 years	39 (29–49)	0.04 (0.03–0.06)	29 (23–37)	0.02 (0.02–0.03)	−2.59 (−2.89 to 2.3)
35–39 years	58 (43–73)	0.06 (0.05–0.08)	36 (29–44)	0.03 (0.03–0.04)	−2.91 (−3.29 to 2.53)
40–44 years	57 (41–72)	0.08 (0.06–0.11)	40 (31–51)	0.04 (0.03–0.06)	−2.81 (−3.16 to 2.47)
45–49 years	56 (40–70)	0.11 (0.08–0.14)	62 (46–80)	0.06 (0.04–0.07)	−2.42 (−2.59 to 2.24)
50–54 years	78 (57–100)	0.16 (0.12–0.21)	95 (73–122)	0.08 (0.06–0.1)	−2.89 (−3.16 to 2.62)
55–59 years	127 (91–161)	0.29 (0.21–0.37)	149 (113–196)	0.14 (0.1–0.18)	−3 (−3.27 to 2.72)
60–64 years	139 (104–175)	0.39 (0.29–0.5)	154 (119–196)	0.21 (0.16–0.27)	−2.47 (−2.67 to 2.27)
65–69 years	172 (126–215)	0.63 (0.46–0.79)	249 (194–318)	0.32 (0.25–0.41)	−2.64 (−2.91 to 2.37)
70–74 years	210 (154–262)	1.12 (0.82–1.39)	333 (264–416)	0.62 (0.5–0.78)	−2.41 (−2.71 to 2.12)
75–79 years	200 (150–251)	1.75 (1.32–2.21)	349 (274–440)	1.05 (0.83–1.33)	−2.08 (−2.33 to 1.84)
80–84 years	145 (104–187)	2.73 (1.97–3.52)	350 (275–457)	1.77 (1.39–2.31)	−1.87 (−2.16 to 1.59)
85–89 years	85 (60–113)	5.07 (3.57–6.67)	335 (263–423)	3.52 (2.76–4.44)	−1.7 (−2.13 to 1.27)
90–94 years	32 (21–44)	10.53 (6.97–14.22)	181 (137–237)	6.19 (4.68–8.07)	−2.26 (−2.69 to 1.83)
95+ years	6 (3–8)	14 (7.65–20)	55 (35–78)	8.55 (5.55–12.15)	−2.3 (−2.74 to 1.86)

EAPC, estimated annual percentage change; UI, uncertainty interval; CI, confidence interval.

**Table 4:** The number of DALYs cases and the age-standardized DALYs rate attributable to non-rheumatic valvular heart disease globally and in China in 1990 and 2021, and its trends from 1990 to 2021 globally.

Region/Subgroup	Number of DALYs cases (95% UI) in 1990	The age-standardized DALYs rate/100,000 (95% UI) in 1990	Number of DALYs cases (95% UI) in 2021	The age-standardized DALYs rate/100,000 (95% UI) in 2021	EAPC (95% CI)
Global	1,791,844 (1,646,002–1,967,041)	49.31 (45.31–54.17)	3,238,185 (2,934,104–3,594,474)	39.72 (35.79–44.14)	−0.66 (−0.75 to 0.57)
Sex in global					
Female	932,933 (824,725–1,045,824)	46.27 (41.14–51.72)	1,681,392 (1,456,551–1,880,870)	36.38 (31.68–40.86)	−0.74 (−0.82 to 0.66)
Male	858,911 (794,324–945,731)	52 (48.1–57.27)	1,556,793 (1,417,075–1,742,183)	43.21 (39.42–48.5)	−0.55 (−0.66 to 0.44)
Age in global					
15–19 years	40,037 (30,390–50,857)	7.71 (5.85–9.79)	42,227 (34,889–49,494)	6.77 (5.59–7.93)	−0.57 (−0.67 to 0.47)
20–24 years	42,329 (32,331–52,192)	8.6 (6.57–10.61)	48,913 (40,918–57,165)	8.19 (6.85–9.57)	−0.39 (−0.5 to 0.28)
25–29 years	44,087 (35,794–53,596)	9.96 (8.09–12.11)	50,328 (43,254–57,664)	8.55 (7.35–9.8)	−0.47 (−0.55–0.38)
30–34 years	45,936 (38,831–54,385)	11.92 (10.07–14.11)	57,559 (50,625–64,462)	9.52 (8.37–10.66)	−0.66 (−0.78 to 0.54)
35–39 years	53,311 (45,912–62,457)	15.13 (13.03–17.73)	69,895 (61,499–78,841)	12.46 (10.97–14.06)	−0.7 (−0.88 to 0.52)
40–44 years	59,876 (52,678–68,778)	20.9 (18.39–24.01)	84,866 (76,449–93,679)	16.96 (15.28–18.73)	−0.91 (−1.12 to 0.7)
45–49 years	64,731 (57,974–74,005)	27.88 (24.97–31.87)	95,608 (86,691–106,519)	20.19 (18.31–22.5)	−1.22 (−1.37 to 1.07)
50–54 years	84,329 (76,478–95,164)	39.67 (35.98–44.77)	121,535 (110,505–131,356)	27.32 (24.84–29.52)	−1.3 (−1.44 to 1.15)
55–59 years	115,916 (105,682–129,301)	62.59 (57.06–69.82)	171,787 (157,061–187,853)	43.41 (39.69–47.47)	−1.13 (−1.33 to 0.93)
60–64 years	151,915 (140,195–165,843)	94.59 (87.29–103.26)	207,177 (190,385–227,050)	64.73 (59.49–70.94)	−1.31 (−1.51 to 1.12)
65–69 years	188,982 (174,787–207,504)	152.89 (141.4–167.87)	267,456 (242,839–301,167)	96.96 (88.04–109.18)	−1.42 (−1.6 to 1.24)
70–74 years	204,626 (188,253–229,086)	241.7 (222.36–270.59)	341,087 (306,786–394,547)	165.71 (149.04–191.68)	−1.36 (−1.53 to 1.2)
75–79 years	247,290 (226,520–276,211)	401.73 (367.99–448.72)	356,144 (318,562–412,600)	270.04 (241.55–312.85)	−1.26 (−1.34 to 1.17)
80–84 years	219,737 (196,776–246,773)	621.15 (556.24–697.57)	418,176 (361,524–479,367)	477.46 (412.78–547.33)	−0.82 (−0.92 to 0.72)
85–89 years	142,410 (123,214–158,630)	942.42 (815.39–1,049.76)	410,396 (339,315–464,910)	897.59 (742.13–1,016.82)	0.05 (−0.11 to 0.21)
90–94 years	64,593 (53,383–72,313)	1,507.36 (1,245.75–1,687.5)	323,443 (252,929–364,767)	1,808.02 (1,413.85–2,039.01)	0.84 (0.7–0.98)
95 + years	21,738 (16,860–24,632)	2,135.18 (1,656.05–2,419.42)	171,588 (124,152–197,560)	3,148.23 (2,277.89–3,624.75)	1.31 (1.18–1.45)
China	58,153 (44,590–71,205)	7.18 (5.6–9.05)	94,587 (73,044–125,926)	4.94 (3.84–6.49)	−1.46 (−1.62 to 1.31)
Sex in China					
Female	24,999 (15,772–33,614)	5.98 (3.93–7.97)	37,094 (26,912–52,185)	3.61 (2.63–5.11)	−1.94 (−2.11 to 1.76)
Male	33,154 (25,496–41,401)	8.73 (6.77–11.23)	57,493 (42,888–77,059)	6.57 (4.95–8.65)	−1.12 (−1.26 to 0.97)
Age in China					
15–19 years	2,989 (2,078–3,802)	2.36 (1.64–3)	614 (475–775)	0.82 (0.64–1.04)	−4.36 (−4.77 to 3.95)
20–24 years	3,450 (2,425–4,345)	2.61 (1.84–3.29)	949 (739–1,252)	1.3 (1.01–1.71)	−3.13 (−3.54 to 2.71)
25–29 years	2,415 (1,717–3,071)	2.2 (1.56–2.79)	1,079 (855–1,355)	1.25 (0.99–1.57)	−2.45 (−2.74 to 2.16)
30–34 years	2,260 (1,660–2,809)	2.56 (1.88–3.18)	1,689 (1,342–2,142)	1.39 (1.11–1.77)	−2.58 (−2.87 to 2.29)
35–39 years	3,079 (2,278–3,863)	3.37 (2.49–4.23)	1,903 (1,525–2,352)	1.8 (1.44–2.22)	−2.89 (−3.27 to 2.51)
40–44 years	2,743 (1,986–3,449)	4.09 (2.96–5.14)	1,951 (1,507–2,477)	2.13 (1.65–2.71)	−2.8 (−3.14 to 2.45)
45–49 years	2,445 (1,768–3,044)	4.74 (3.42–5.9)	2,738 (2,034–3,528)	2.48 (1.84–3.2)	−2.38 (−2.55 to 2.21)
50–54 years	3,109 (2,230–3,965)	6.52 (4.67–8.31)	3,889 (2,993–4,962)	3.22 (2.48–4.11)	−2.78 (−3.04 to 2.52)
55–59 years	4,630 (3,372–5,736)	10.68 (7.78–13.23)	5,782 (4,386–7,543)	5.26 (3.99–6.86)	−2.78 (−3.05 to 2.52)
60–64 years	4,942 (3,660–6,264)	13.99 (10.36–17.73)	6,104 (4,666–8,114)	8.36 (6.39–11.11)	−2.05 (−2.22 to 1.89)
65–69 years	6,230 (4,575–8,208)	22.84 (16.77–30.08)	11,578 (8,381–16,255)	15.09 (10.93–21.19)	−1.59 (−1.75 to 1.43)
70–74 years	7,420 (5,561–10,119)	39.43 (29.55–53.77)	15,979 (11,436–22,958)	29.98 (21.46–43.08)	−1.02 (−1.13 to 0.9)
75–79 years	6,422 (4,749–8,949)	56.43 (41.73–78.64)	15,274 (10,817–21,997)	46.12 (32.66–66.42)	−0.63 (−0.71 to 0.55)
80–84 years	3,818 (2,821–5,357)	72.08 (53.26–101.13)	12,402 (8,999–17,945)	62.66 (45.47–90.67)	−0.46 (−0.55 to 0.38)
85–89 years	1,660 (1,260–2,217)	98.39 (74.68–131.43)	8,211 (6,038–11,231)	86.2 (63.39–117.9)	−0.54 (−0.71 to 0.36)
90–94 years	460 (346–596)	149.87 (112.81–194.17)	3,366 (2,575–4,430)	114.8 (87.84–151.1)	−1.06 (−1.26 to 0.86)
95+ years	81 (59–106)	200.55 (145.87–262.03)	1,079 (810–1,423)	168.85 (126.75–222.62)	−0.86 (−1.06 to 0.66)

EAPC, estimated annual percentage change; UI, uncertainty interval; CI, confidence interval.

China exhibited distinct epidemiological patterns. In 2021, incidence cases numbered 292,215 (95% UI: 275,208–310,319), with an ASIR of 12.75 per 100,000 (95% UI: 12.01–13.54). Prevalence cases totaled 3,277,574 (95% UI: 3,093,730–3,482,108), corresponding to an ASPR of 156.24 per 100,000 (95% UI: 147.74–165.63). Deaths (2,458; 95% UI: 1,952–3,079) and DALYs (94,587; 95% UI: 73,044–125,926) were markedly lower than global averages, with ASDR and ASDAR at 0.13 (95% UI: 0.11–0.17) and 4.94 (95% UI: 3.84–6.49) per 100,000, respectively (Tables 1–4).

**Figure 1:**
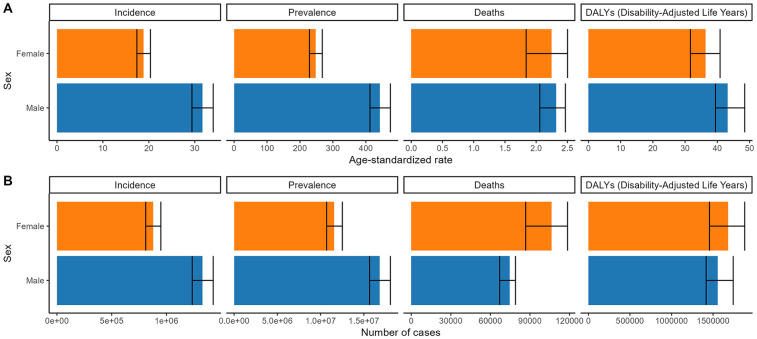
Numbers and age-standardized rates of non-rheumatic valvular heart disease related incidence, prevalence, deaths, and DALYs for both sex in 2021 globally. **(A)** Age-standardized rate. **(B)** Number of cases.

**Figure 2:**
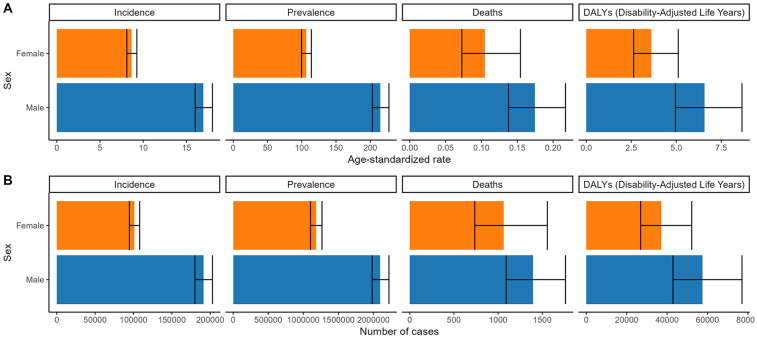
Numbers and age-standardized rates of non-rheumatic valvular heart disease related incidence, prevalence, deaths, and DALYs for both sex in 2021 in China. **(A)** Age-standardized rate. **(B)** Number of cases.

Globally, males exhibited higher burden metrics than females for incidence, prevalence, deaths, DALYs cases and the corresponding ASRs, except for ASDR and ASDAR. In contrast, Chinese males consistently surpassed females across all indicators (Figures 1, 2 and Tables 1–4).

**Figure 3:**
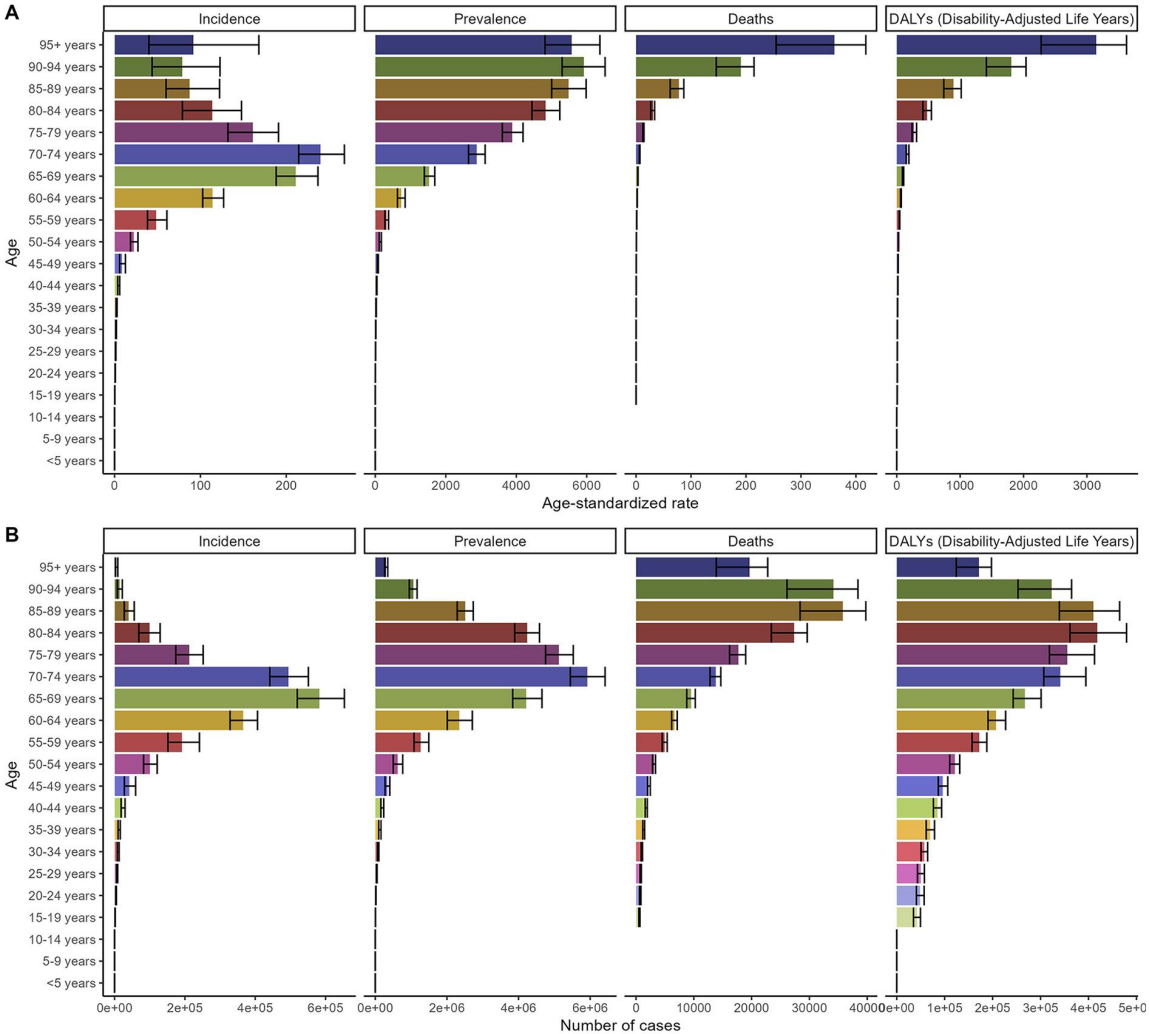
Numbers and age-standardized rates of non-rheumatic valvular heart disease related incidence, prevalence, deaths, and DALYs for different age groups in 2021 globally. **(A)** Age-standardized rate. **(B)** Number of cases.

**Figure 4:**
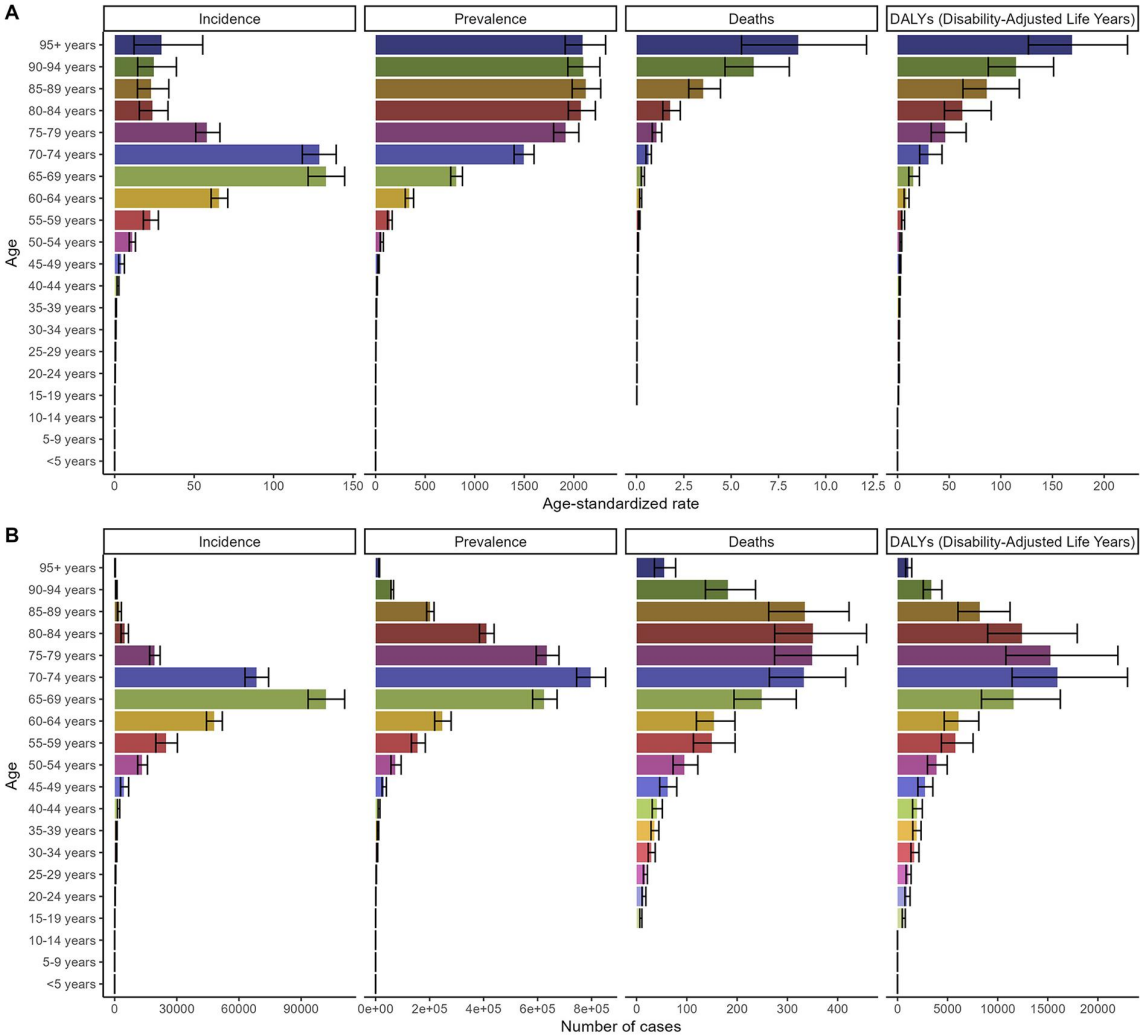
Numbers and age-standardized rates of non-rheumatic valvular heart disease related incidence, prevalence, deaths, and DALYs for different age groups in 2021 in China. **(A)** Age-standardized rate. **(B)** Number of cases.

The distribution of incidence, prevalence, deaths, and DALYs across age groups globally and in China in 2021 is presented in Figures 3, 4. The number of incidence, prevalence, deaths, and DALYs cases initially increased with age, reaching a peak and then declining globally and in China. The ASDR and ASDAR continuously increased with age. However, ASIR demonstrated a bimodal age distribution: an initial rise in young adulthood, a mid-life decline, and a secondary peak after age 95 (Figures 3, 4 and Tables 1–4).

### 3.2 Temporal trends in disease burden: 1990–2021

**Figure 5:**
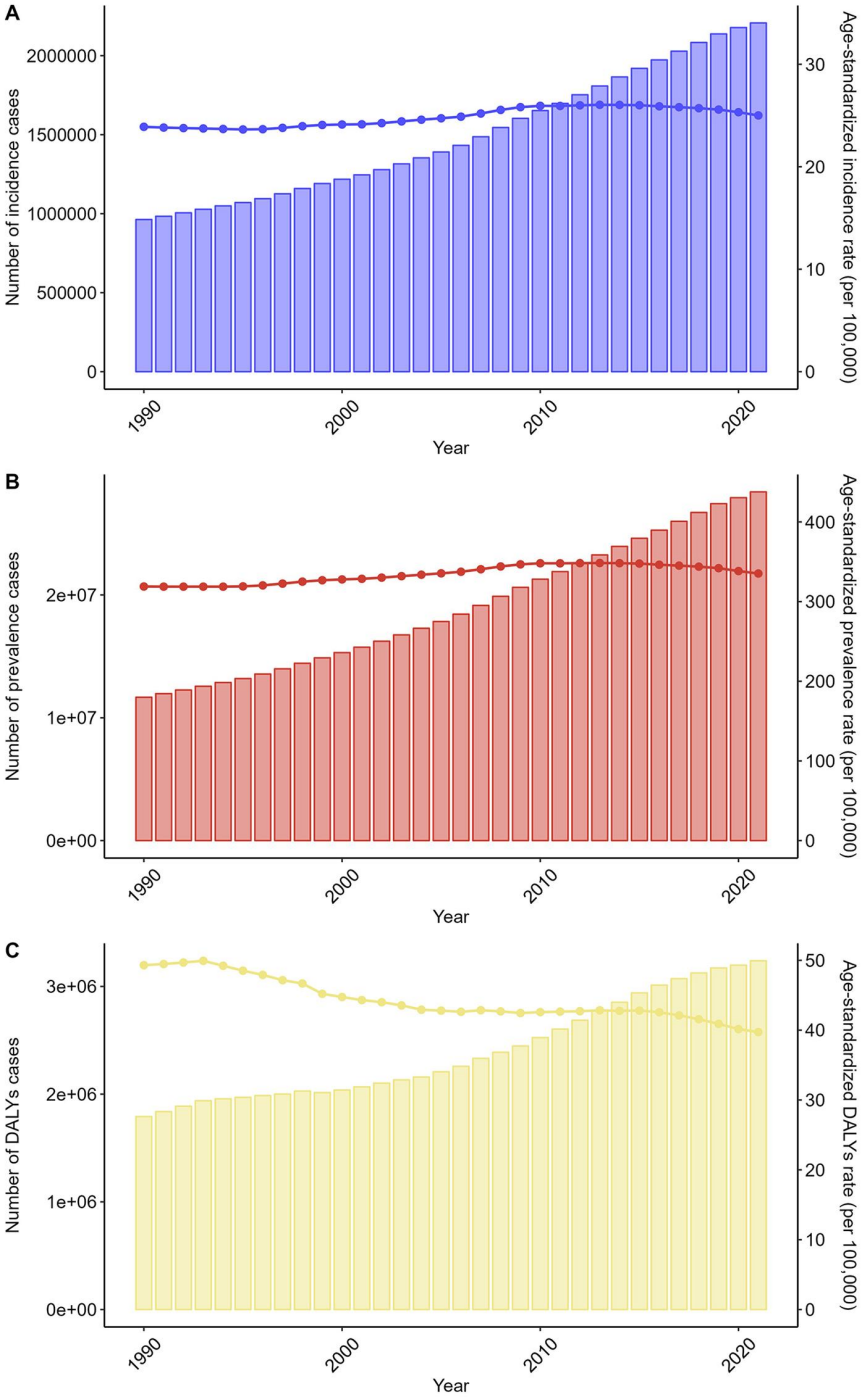
Trends in the numbers and age-standardized rates of non-rheumatic valvular heart disease-related incidence, prevalence, and DALYs globally from 1990 to 2021. **(A)** Number of incidence cases. **(B)** Number of prevalence cases. **(C)** Number of DALYs cases.

**Figure 6:**
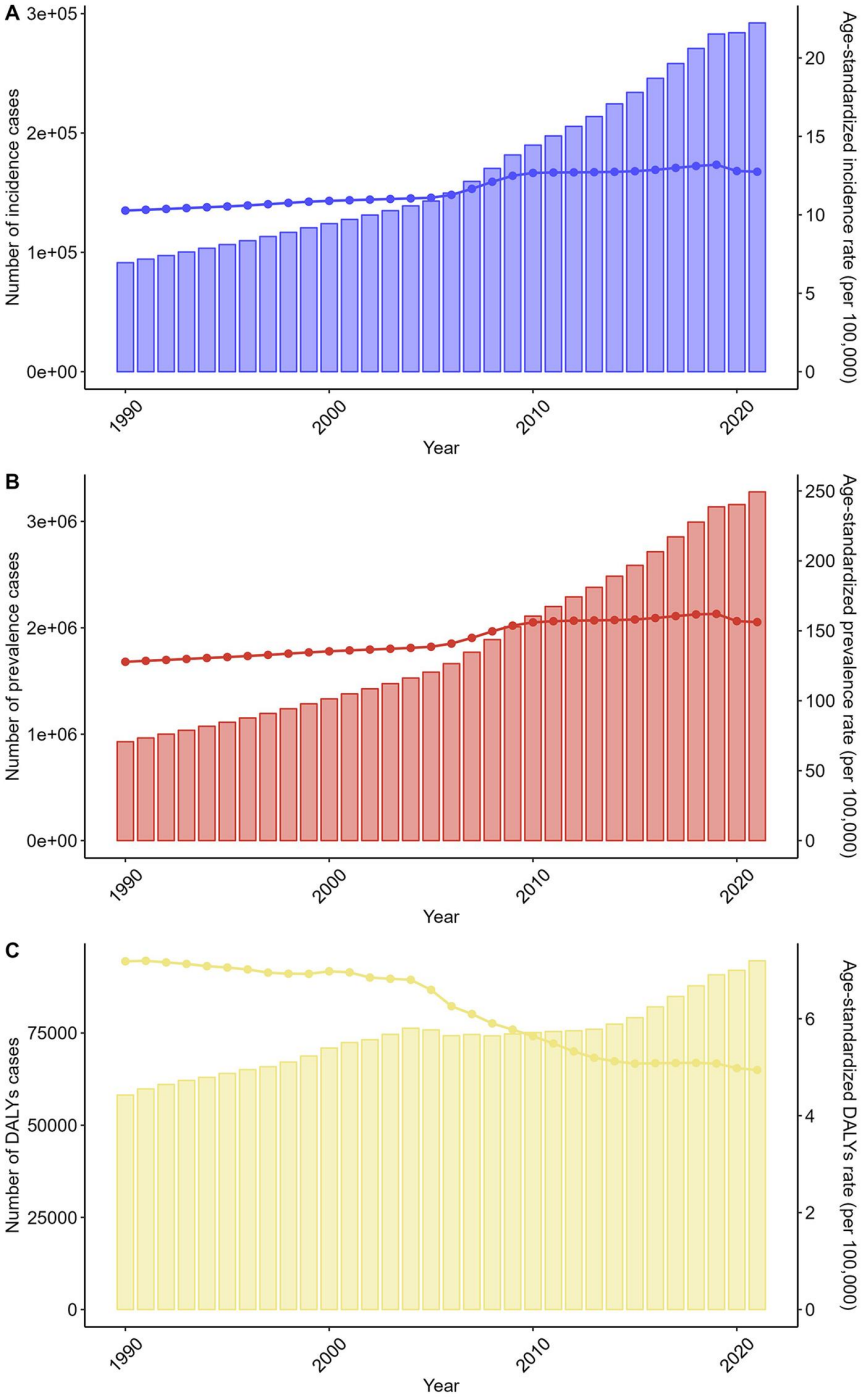
Trends in the numbers and age-standardized rates of non-rheumatic valvular heart disease-related incidence, prevalence, and DALYs in China from 1990 to 2021. **(A)** Number of incidence cases. **(B)** Number of prevalence cases. **(C)** Number of DALYs cases.

Between 1990 and 2021, NRVHD burden escalated globally. incidence cases increased by 129.1% (963,242–2,206,928), while prevalence nearly tripled (6,729,808 to 28,389,034). Deaths and DALYs rose by 119.2% (82,630–181,078) and 80.8% (1,791,844–3,238,185), respectively. Age-standardized rates revealed divergent trends: ASIR increased from 23.9 to 25.0 per 100,000 (+4.6%), and ASPR rose from 422.15 to 442.45 per 100,000 (+4.8%). Conversely, ASDR and ASDAR declined by 13.2% (2.66–2.31) and 19.5% (49.31–39.72), respectively (Figures 5, 6 and Tables 1–4).

China mirrored global patterns but with amplified growth. incidence cases surged by 220.1% (91,321–292,215), and prevalence increased 3.5-fold (929,466–3,277,574). Deaths and DALYs rose by 60.1% (1,535–2,458) and 62.6% (58,153–94,587). Age-standardized metrics showed similar divergence: ASIR increased from 10.27 to 12.75 per 100,000 (+24.1%), and ASPR rose from 127.84 to 156.24 per 100,000 (+22.2%). ASDR and ASDAR declined by 43.5% (0.23–0.13) and 31.2% (7.18 to 4.94), respectively (Figures 5, 6 and Tables 1–4).

Sex-stratified analyses confirmed parallel trends in males and females globally and in China (Supplementary Figures S1, S2 and Tables 1–4). Additionally, the trends were also consistent across all age groups globally and in China (Supplementary Figures S3 and S4 and Tables 1–4).

## 4 Discussion

This systematic analysis of GBD 2021 data reveals three critical epidemiological transitions in NRVHD: (1) a global escalation in incidence and prevalence contrasting with declining mortality rates, (2) accelerated burden growth in China surpassing global averages, and (3) persistent sex-age disparities shaped by demographic and healthcare inequities. These findings align with, yet crucially extend, prior research on VHD epidemiology, while exposing unique challenges for health systems navigating aging populations and evolving disease profiles.

The observed 129.1% global increase in NRVHD incidence (1990–2021) mirrors population aging trajectories, particularly in high-income countries where degenerative aortic stenosis prevalence triples with each decade beyond age 65 (14). Our ASPR increase (+4.8%) corroborates Coffey et al.'s projection that non-rheumatic etiologies now account for >70% of VHD cases in aging societies (15). Moreover, diagnostic drift must be considered: increased echocardiography access in LMICs [5-fold since 2000 (20)] amplified detection of subclinical cases, partially explaining the incidence-prevalence-mortality divergence. However, the concurrent 13.2% ASDR decline challenges historical assumptions about NRVHD's intractability. Therapeutic advances contributed significantly, particularly in China where TAVI expansion [100 to 6,500 procedures/year, 2012–2021 (28)] contributed to the ASDR decline.

Advancements in minimally invasive interventions, notably transcatheter aortic valve implantation (TAVI), have expanded treatment eligibility. Since its 2002 introduction, TAVI utilization has grown exponentially, with 150,000 annual procedures globally by 2021 (16). This technological shift reduced perioperative mortality from 8.2% (surgical AVR) to 2.9% (TAVI) in high-risk patients (17), partially explaining mortality declines despite rising case numbers.

Second, improved cardiovascular risk factor management has altered disease progression. The 19.5% global ASDAR reduction aligns with studies showing statins and antihypertensives slow calcific aortic stenosis progression by 18%–34% (18). In China, aggressive hypertension control (prevalence reduced from 21.7% to 15.3% in 1990–2021) (19) may have contributed to its steeper 31.2% ASDAR decline.

Third, diagnostic ascertainment bias cannot be discounted. Echocardiography availability increased 5-fold in low/middle-income countries (LMICs) since 2000 (20), potentially inflating incidence counts through detection of subclinical cases. This “diagnostic creep” may artificially amplify morbidity-mortality divergence, a phenomenon previously documented in chronic kidney disease epidemiology (21).

China's 220.1% NRVHD incidence surge, triple the global rate, epitomizes the collision between rapid aging and incomplete healthcare modernization. Three factors dominate:

1. Demographic Pressures: China's ≥65 population ballooned from 5.6% (1990) to 13.5% (2021) (22), directly fueling degenerative valve disease. Our estimated ASPR of 156.24/100,000 aligns with Wu et al.'s national survey finding aortic stenosis prevalence tripling among those ≥75 years (2001–2015) (23).
2. Epidemiological Transition: As China controlled rheumatic fever (RHD prevalence dropped 73% since 1990) (24), NRVHD emerged as the dominant valvulopathy. Hospitalization data shows degenerative VHD surpassing RHD as the leading valve surgery indication since 2010 (25).
3. Healthcare Access Gaps: Despite progress, echocardiography penetration remains uneven, 85% in urban vs. 32% in rural China (26). This underdiagnosis paradoxically suggests our estimates may understate true burden, particularly in western provinces.

However, China's mortality decline (−43.5% ASDR) outpaces global trends, likely reflecting concentrated investment in tertiary cardiac care. Between 2015 and 2021, China increased cardiac surgery centers from 564 to 1,122 (27), with TAVI procedures growing from 100 (2012) to 6,500 annually (28). Yet this progress remains fragile: rural mortality rates are 3.1-fold higher than urban areas for valve diseases (29), exposing persistent equity challenges.

Our findings reinforce, but complicate, established narratives about VHD disparities. Globally, males’ higher incidence/prevalence aligns with known sex-specific risk profiles: aortic stenosis progression is 40% faster in males (30), while hypertension, a key mitral regurgitation driver, affects 31% of men vs. 26% of women globally (31). However, the absence of sex differences in ASDR/ASDAR contradicts studies suggesting worse post-intervention survival in women (32). This paradox may reflect:

1. Treatment Bias: Women are 23% less likely to undergo valve surgery at advanced ages (33), potentially excluding high-risk females from mortality statistics.
2. Competing Risks: Longer female life expectancy increases non-cardiac mortality, diluting NRVHD-specific death rates.

In China, males’ across-the-board burden dominance likely intertwines biological and cultural factors. Smoking rates are 48.4% in men vs. 2.0% in women (34), exacerbating aortic calcification. Simultaneously, patriarchal norms may prioritize male healthcare access, a phenomenon observed in cancer screening disparities (35).

Age distribution patterns reveal another layer of complexity. The bimodal ASIR peak, rising in young adulthood (congenital/residual RHD), dipping mid-life, then surging post-70 (degenerative), mirrors pathophysiological transitions documented in European registries (36). Age distribution patterns further revealed clinical imperatives: bimodal ASIR peaks (young adulthood and senescence) underscore the need for early-life congenital/residual RHD screening and age-targeted degenerative disease surveillance. The steep elderly incidence spike suggests delayed diagnosis: median time from symptom onset to surgery remains 5.2 years vs. 1.8 years in the U.S (37).

These findings necessitate tiered policy responses:

Global Priorities:

1. Enhanced Surveillance: Current ICD coding often conflates valve etiologies (38). WHO should mandate distinct NRVHD/RHD reporting to guide resource allocation.
2. TAVI Cost-Reduction: With 80% of LMICs lacking TAVI access (39), device cost-innovation (China's Venus Valve priced at 12,000 vs.12,000 vs.32,000 Western equivalents) (40) is critical.

China-Specific Strategies:

1. Primary Prevention: Integrate valve screening into national hypertension/diabetes programs, a model piloted in Guangdong reduced severe VHD by 18% (41).
2. Regional Resource Balancing: Expand “mobile echo units” to rural areas, replicating Sichuan Province's 37% case-detection increase (42).
3. Sex-Sensitive Care: Address implicit bias in treatment referrals through clinician training programs.

While leveraging GBD's standardized methodology, several limitations warrant consideration:

1. Etiological Overlap: GBD's reliance on hospital data may undercount “secondary” NRVHD (mitral regurgitation from ischemic cardiomyopathy) (43).
2. Treatment Effect Lag: Mortality declines from recent TAVI adoption may be underestimated in 2021 data.
3. Chinese Subtypes: Lack of provincial-level etiology data (bicuspid aortic valve prevalence varies from 0.5% in Guangxi to 1.2% in Shandong) (44) limits localized interventions.

Future research should prioritize etiology-specific burden mapping and cost-effectiveness analyses of novel interventions across diverse healthcare settings.

This study has several methodological constraints. First, reliance on modeled GBD estimates rather than primary population-based registries may mask subnational heterogeneities, particularly given the absence of provincial-level data for China. Consequently, localized burden patterns (e.g., rural-urban gradients) remain incompletely characterized. Second, aggregation of non-rheumatic valvular heart disease without etiological stratification (e.g., aortic stenosis *vs.* mitral regurgitation) limits mechanistic insights and precludes lesion-specific interventions. Third, potential misclassification of secondary valvular lesions—notably ischemic mitral regurgitation—may occur in administrative coding systems, affecting burden accuracy. Finally, while mortality declines were statistically robust globally, wider UIs in under-surveilled regions indicate reduced precision in subnational estimates due to sparse data. These limitations underscore the need for enhanced clinical registries to validate model outputs and capture etiological spectra.

## 5 Conclusion

This systematic analysis of GBD 2021 data highlights the dual challenge of rising NRVHD morbidity and declining mortality globally and in China, driven by population aging and epidemiological transition. Between 1990 and 2021, global NRVHD incidence surged by 129%, with China experiencing a 220% increase, reflecting accelerated demographic shifts. While age-standardized mortality rates declined by 13.2% globally and 43.5% in China, attributable to advances in transcatheter interventions and cardiovascular risk management, persisting sex disparities (male predominance) and geographic inequities (rural China's mortality tripling urban rates) underscore unmet needs. China's rapid burden escalation, juxtaposed with fragmented healthcare access, calls for prioritized primary prevention (hypertension control), equitable diffusion of cost-effective technologies (domestically produced TAVI valves), and strengthened surveillance systems to address aging-related valvulopathies. These findings mandate integrated strategies balancing therapeutic innovation with preventive equity to mitigate NRVHD's growing societal impact.

## Data Availability

The original contributions presented in the study are included in the article/Supplementary Material, further inquiries can be directed to the corresponding authors.
