# Supplementary material for: Burden of non-rheumatic valvular heart disease globally and in China from 1990 to 2021: a systematic analysis for the global burden of disease study 2021
Source: Front Cardiovasc Med. 2025 Nov 20;12:1641448. doi: 10.3389/fcvm.2025.1641448 (PMC12675378; doi:10.3389/fcvm.2025.1641448)

Supplementary Information and Data

Burden of non-rheumatic valvular heart disease globally and in China from 1990 to 2021: a systematic analysis for the Global Burden of Disease Study 2021

Supplementary Figures

Figure S1. Trends in the numbers and age-standardized rates of non-rheumatic valvular heart disease-related incidence, prevalence, deaths, and DALYs globally by sex from 1990 to 2021.

Figure S2. Trends in the numbers and age-standardized rates of non-rheumatic valvular heart disease-related incidence, prevalence, deaths, and DALYs in China by sex from 1990 to 2021.

Figure S3. Trends in the numbers and age-standardized rates of non-rheumatic valvular heart disease-related incidence, prevalence, deaths, and DALYs globally by age groups from 1990 to 2021.

Figure S4. Trends in the numbers and age-standardized rates of non-rheumatic valvular heart disease-related incidence, prevalence, deaths, and DALYs in China by age groups from 1990 to 2021.

Figure S1. Trends in the numbers and age-standardized rates of non-rheumatic valvular heart disease-related incidence, prevalence, deaths, and DALYs globally by sex from 1990 to 2021.

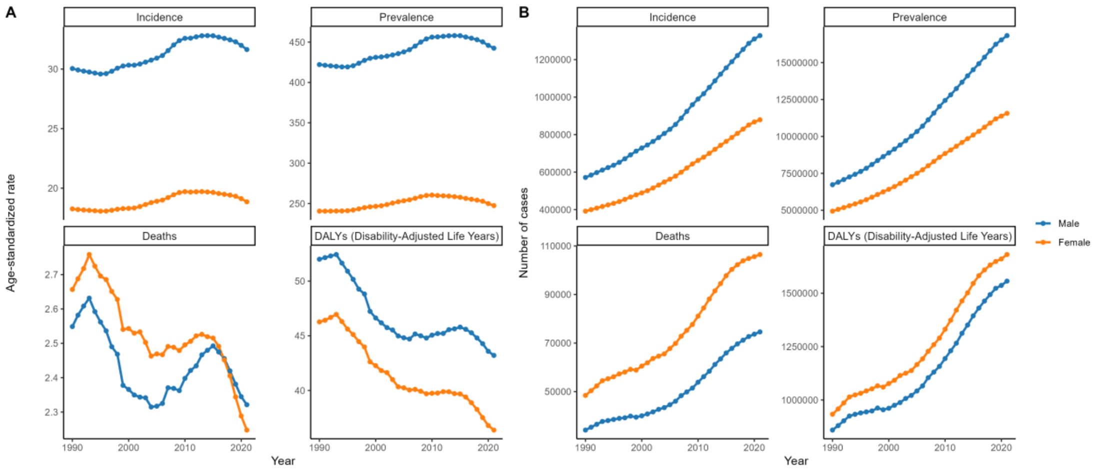

Figure S2. Trends in the numbers and age-standardized rates of non-rheumatic valvular heart disease-related incidence, prevalence, deaths, and DALYs in China by sex from 1990 to 2021.

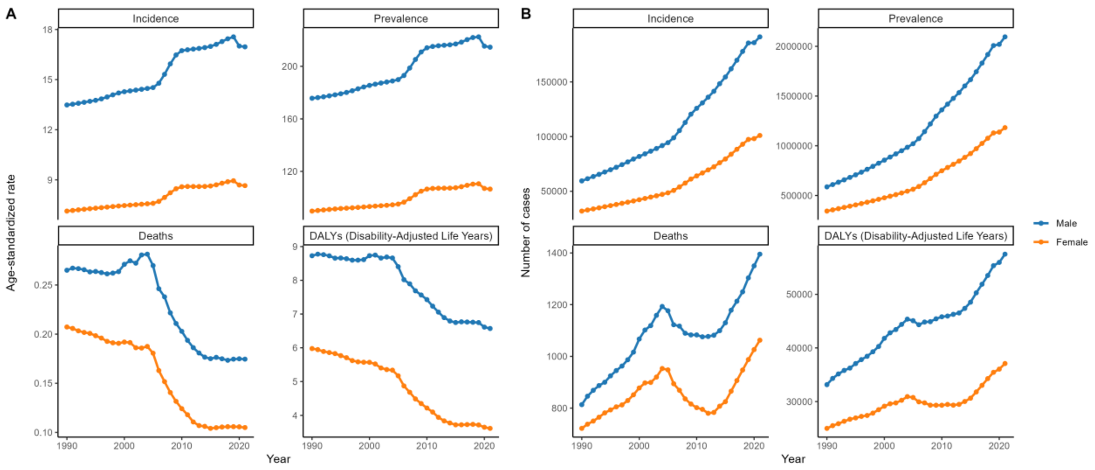

Figure S3. Trends in the numbers and age-standardized rates of non-rheumatic valvular heart disease-related incidence, prevalence, deaths, and DALYs globally by age groups from 1990 to 2021.

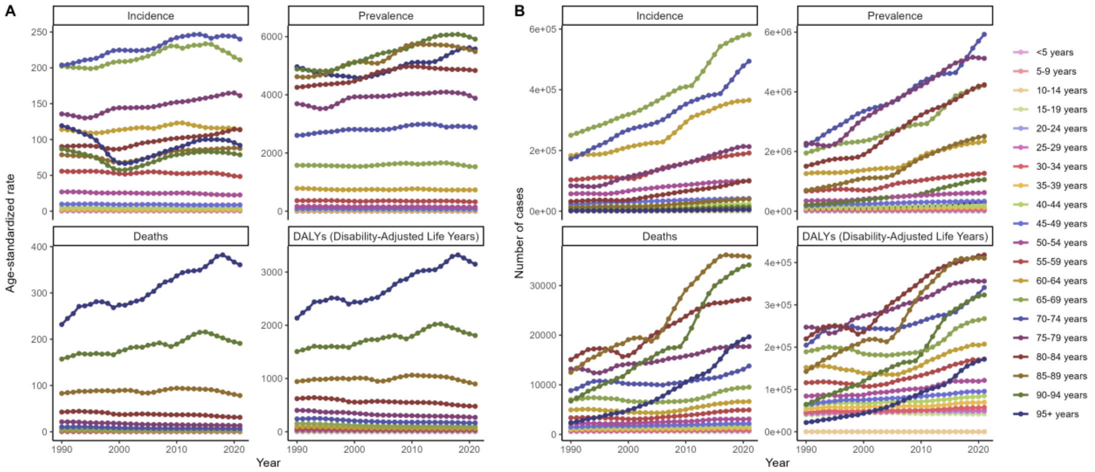

Figure S4. Trends in the numbers and age-standardized rates of non-rheumatic valvular heart disease-related incidence, prevalence, deaths, and DALYs in China by age groups from 1990 to 2021.

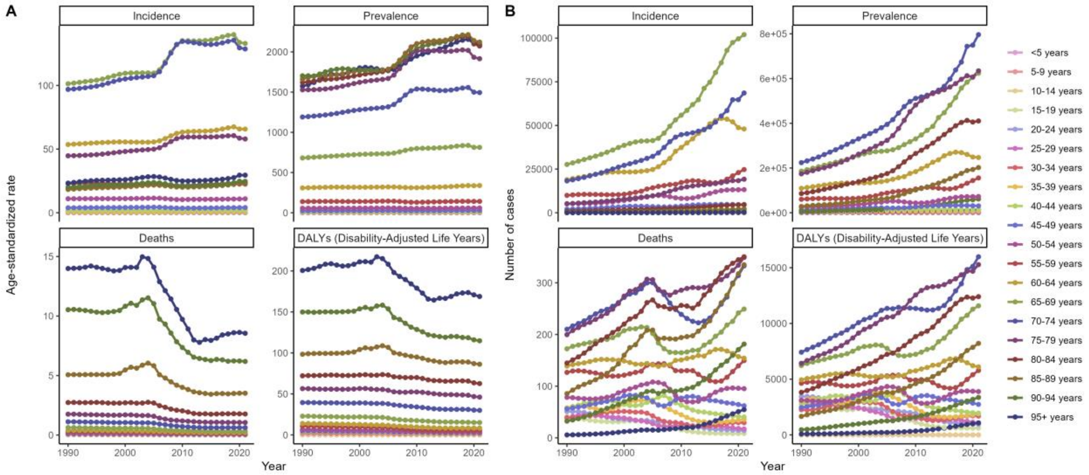

Supplement: Supplementary file 1 [file DataSheet1.pdf]
